# Piceatannol induced apoptosis through up-regulation of microRNA-181a in melanoma cells

**DOI:** 10.1186/s40659-017-0141-8

**Published:** 2017-10-17

**Authors:** Maotao Du, Zhong Zhang, Tao Gao

**Affiliations:** 1 Department of Dermatology, The Second Affiliated Hospital of Chongqing Medical University, 76 Linjiang Road, Yuzhong District, Chongqing, 400010 China; 2 Department of Dermatology, Chongqing Traditional Chinese Medicine Hospital, Chongqing, 400021 China

**Keywords:** Piceatannol, Apoptosis, microRNA-181-a, Melanoma cells

## Abstract

**Background:**

Melanoma took top position among the lethal cancers and, despite there have been some great attempts made to increase the natural life of patients with metastatic disease, long-lasting and complete remissions are few. Piceatannol, owns the similar function as resveratrol, has been defined as an anti-cancer agent playing important role in inhibition of proliferation, migration and metastasis in various cancer. Thus, we aim to investigate the anti-cancer effect and mechanisms of piceatannol in melanoma cells.

**Methods:**

Melanoma cell lines WM266-4 and A2058 were treated either with or without piceatannol. Cell viability and cell apoptosis were assessed by using MTT and Annexin V/PI assay, respectively. Cells were transfected with specific miRNA using Lipfectamine 2000. miRNA bingding ability to 3'-UTR region within specific gene was assed by firefly luciferase analysis. Gene and protein expression was eveluated by qRT-PCR and western blot analysis, respectively.

**Results:**

Our study showed that piceatannol inhibited WM266-4 and A2058 cells growth and induced apoptosis. Totally, 16 differentially expressed miRNAs were screened out including 8 up-regulated and 8 down-regulated miRNAs. Expression level of miR-181a is significantly higher in piceatannol-treated cells than normal control and is lower in melanoma cancer tissues than its adjacent normal tissues. Bcl-2 is a target gene of miR-181a. Moreover, silencing of miR-181a reverses the decrease of cell viability induced by piceatannol in WM266-4 and A2058 cells. Taken together, present study uncovered the ability of piceatannol to repress melanoma cell growth and clarified the contribution of miR-181a in the anticancer role of piceatannol.

**Conclusion:**

The present study proposes that piceatannol can be taken into account to be a hopeful anticancer agent for melanoma.

## Background

Melanoma is aggressive skin cancer and its incidence continues to increase globally [1, 2]. It is the fifth and sixth most common cancer in males and females, respectively [3], and is one of the most common cancers among adolescents and young adults [4]. It is known that surgical resection is a promising and curative intervention when melanoma is diagnosed at early stage, but many patients show up with unresectable cancer at later stages which is usually treated with surgical operation, commonly together with adjuvant chemotherapy [5]. By reason of its complex etiology addition to drug/chemo resistance and high metastatic potential, poor prognosis often present in patients who are diagnosed with advanced melanoma, remaining therapeutically unsatisfactory for many years [6]. For cancer chemo prevention or treatment, the discovery and development of novel medicament with low toxicity, high efficiency and excellent potential is an important part of cancer therapies. Thus, it is needed to find effective bio-compound by studying its molecular mechanism at the basis of melanoma pathogenesis in order to provide potential targets for its diagnosis and treatment.

Piceatannol is a strong antioxidant and owns anticancer and chemo preventive abilities. Despite the pharmacological actions of piceatannol, especially its antioxidant, antitumor, and anti-inflammatory activities, including bioavailability and toxicity in humans are well documented, depending on the cell type piceatannol may either stimulate [7, 8] or inhibit [7] apoptosis. Although it was reported that the evaluation of anti-melanoma effect of piceatannol on human melanoma cells SK-Mel-28 [9], the results limited with supporting metabolic data and therapeutic effects and underlying mechanism of piceatannol on human melanoma remains unknown. Thereby, these limitations are attempted to be replenished in our study.

It is the fact that microRNAs (miRs) are a class of small non-coding RNA that can specifically binding to the 3′-untranslational region of their target mRNAs and in this way cause mRNA degradation or inhibition of protein translation [10, 11]. Altered miRNA expression has been widely reported in cancer, it has been reported that high levels of oncogenic miRNAs downregulate tumor suppressor genes, and conversely, decreased tumor suppressor miRNAs responsible for elevated oncogene expression [12]. By means of regulating the expression of their target genes, miRNAs affect diverse of biological processes, such as cell proliferation, survival, differentiation, cell cycle progression and apoptosis [13]. In addition to their prognostic or diagnostic utility as biomarkers, miRNAs have appeared as novel biological tool for cancer treatment [14–16]. It is complex to escape from apoptosis for cancer cells. Highly expressed levels of anti-apoptotic protein, B cell lymphoma 2 (Bcl-2) is known to be one of the major causal factors in tumorigenesis. In the apoptotic process of eukaryotic cells, Bcl-2 plays an crucial role contributing survival in inhibition of cell death [17]. Highly expressed level of Bcl-2 has been reported in various cancers including melanoma [18], and has been broadly linked to its anti-apoptotic activity in melanoma cells, silencing by miRNAs [19].

In present study, we attempt to investigate the anti-cancer mechanisms of piceatannol in the highly aggressive human melanoma cell line, WM266-4 and A2058, and elucidate the inhibitory effect of piceatannol on melanoma cells through attenuating miRNA-mediated Bcl-2.

## Methods

### Materials and samples

Piceatannol with purity > 99% was obtained from Chengdu Must Bio-tech, and 100 mM stock solution was prepared by dissolving the piceatannol in ethanol and stored at − 20 °C in the dark. MTT based Cell Counting Kit-8 (CCK-8) (Beyotime, Haimen, China), 10% fetal bovine serum (Gibco, UK), antibodies of Bcl-2, Bax, caspase-3 and β-actin were provided from Abcam (Cambridge, MA).

Tissue specimens were obtained from patients who are underwent melanoma surgical resection in the Second Affiliated Hospital of Chongqing Medical University and adjacent tissues, which is classified as control group, were acquired from resected neighboring area of melanoma. We surgically procured tumor samples from patients with primary cutaneous melanoma. Upon surgical removal of the primary melanoma, a single surgical oncologist (A.I.R.) used a scalpel to macrodissect and procured a portion of the remaining primary tumor. We’ve collected 10 paired tissues, namely 10 normal tissues and 10 tumor tissues, in which the level of miR-181a was measured. No patients underwent cutaneous radiotherapy or chemotherapy before. This study was approved by ethics review board of Chongqing Medical University and all the patients provided written informed consent.

### Cell line, culture and piceatannol treatment

Melanoma cells WM266-4 and A2058 were provided from Wuhan cell bank of Procell. Cells were cultured in DMEM contains 10% fetal bovine serum, 100 μg/ml of penicillin and streptomycin, and incubated in a humidified condition of 95% air and 5% CO_2_ at 37 °C. After 24 and 48 h of culturing, the cells treated with piceatannol were collected for following measurement.

### MTT cell viability assay

The 96-hole culture plates was used to seed WM266-4 and A2058 cells with 1 × 10 [4] cells/well, and the plate was placed in incubation box at 37 °C with 5% CO_2_. The cells were treated with different concentrations of piceatannol and after 24-h culture, MTT assay (Gibco) was performed to determine the cell apoptosis. The culture media were placed and 20 μl MTT assay (5 mg/ml) was added to each well prior to 4 h incubation. 150 μl DMSO were added to dissolve the formazan crystals. After 10 min oscillation, the optical density (OD) values were measured with a microplate reader (Beckmann Coulters) at 490 nm. Cell viability was presented as a percent of MTT reduction in the treated cells versus the controls (cells incubated in serum-free DMEM without extracts). The relative MTT level (%) was calculated as [A]/[B] × 100, where [A] is the absorbance of the test sample and [B] is the absorbance of control sample containing the untreated cells. Decreased relative MTT level indicates decreased cell viability.

### Cell apoptosis detection by flow cytometry

In brief, WM266-4 and A2058 cells were harvested and washed with PBS after treatment. The density of cells was adjusted to 3 × 10 [5], then added 400 μl binding buffer, 5 μl Annexin V (Gibco), 10 μl PI (Bio-Science, Shanghai, China), and placed the plate at room temperature for 15 min in the dark. Flow cytometry (LSRII, BD Biosciences) was used to analyze all the samples. Statistical analysis of flow cytometry-obtained apoptosis (%) in both WM266-4 and A2058 cell lines was performed using t test to generate *P* value in order to compute the difference between treated and untreated scores. Statistical analysis between untreated and control and between treated and control was also presented.

### Western blot analysis

The total proteins were extracted from WM266-4 and A2058 cells. Cells were lysed in RIPA buffer (Beyotime Institute of Biotechnology, Haimen, China) for 40 min. 50 μg proteins were separated using 15% SDS–GAGE. After cell supernatants were transfered to new tubes, the protein concentration was measured. The proteins were loaded to gel prior to electrophoresis. Then, the protein was transferred to Nylon membrane. Before the membrane was probed with primary antibodies, the membrane was blocked with 5% nonfat dried milk. Antibody for Bax, Bcl-2 and caspase-3 were purchased from Abcam. The relative protein expression was detected using Image–Pro Plus software, version 6.0 (Media Cybernetics, Inc., Rockville, MD, USA). The protein expression was presented as density ratio vs β-actin. The β-actin antibody (Abcam) was used as an internal control. Levels of all proteins were normalized to respective actin control. The control condition was normalized to 1 and all other experimental conditions were compared to this. All the experiments repeated 5 times and one-way ANOVA was performed for statistical analysis.

### Transfection

Before transfection, WM266-4 and A2058 cells were seeded in 25 cm [2] culture flasks in completed DMEM medium. 500 μl of Opti-MEM Reduced Serum Medium (Thermo Fisher Scientific, Inc) was separately added to the miRNA and lipofectamine 2000 (Thermo Fisher Scientific, Inc), and they were mixed for 5 min. Then, mix these two mixtures into a flask and incubated for 20 min at room temperature. The mixture was added to the cells prior to incubation for 36 h for further experiments.

### Quantification of miRNA expression

Total RNA was extracted from WM266-4 and A2058 cells by TRIZOl_Reagent (Life Technologies, Thermo Fisher Scientific, Inc) following the manufacturer’s protocol. Expression of 16 miRNAs was quantified by PCR array assays purchased from QIAGEN. This array experimentally verified representative miRNAs which regulate various apoptosis related genes, and their inhibition or overexpression resulted in stimulate or attenuate apoptosis. In our analysis to screen differentially expressed miRNAs between the dug treated groups and negative control group, Limma package in R language was used. To calculate the false discovery rate of P value (FDP) and conduct multi-test, the Benjamin-Hochberg’ method was used. Differentially expressed miRNAs were identified with the *P* value < 0.05 and fold change > 1.2 or < 0.8. Then the qualified miRNAs were classified as up-regulated and down-regulated groups.

### Real time PCR detection of miR-181a

To detect transcriptional level of candidate miRNA, 0.5 μl of the product from RT reaction of miR-181a (diluted 1:2) was combined with 0.5 μl of a 20X TaqMan MicroRNA Assay, which includes probe, reverse primer, forward primer, and 5 μl of 2X TaqMan Universal PCR Master Mix to final reaction volume of 20 μl. The expression of miRNA was then determined with the standard TaqMan microRNA assays using Bio-RAD CFX96-Realtime PCR System. Protocol cycling conditions of 95 °C for 10 min, and then, 40 cycles of denaturation at 95 °C for 15 s and annealing step at 60 °C for 60 s. After normalization to U6, alteration in expression level of miR-181a between different samples were calculated. The one-way ANOVA was used for statistical analysis. All the *P* values are derived from testing the null hypothesis that ΔΔCt are equal to 0. Therefore, a small *P* value indicates that the ΔΔCt is significantly different from 0, which demonstrates a significant effect. *P* < 0.05 is considered as significant difference.

### RNA hybrid algorithms and firefly luciferase activity

We have identified a putative miR-181a-binding site within the 3'-UTR of Bcl-2 mRNA using Target Scan and RNA hybrid algorithms. The luciferase activity was measured prior to co-transfection of cloned plasmid, with either miR-181a or negative control miRNA, into HEK cells.

### Statistical analyses

All statistical analysis was performed using SPSS software 19.0 version and data were expressed as mean ± SD. A two-tailed *P* < 0.05 was considered as statistically significant. Student’s unpaired t test was used for determining the differences between two group comparison and for more than two group comparisons One-way ANOVA test was used.

## Results

### Piceatanal induces growth and apoptosis of melanoma cells WM266-4 and A2058

**Fig. 1:**
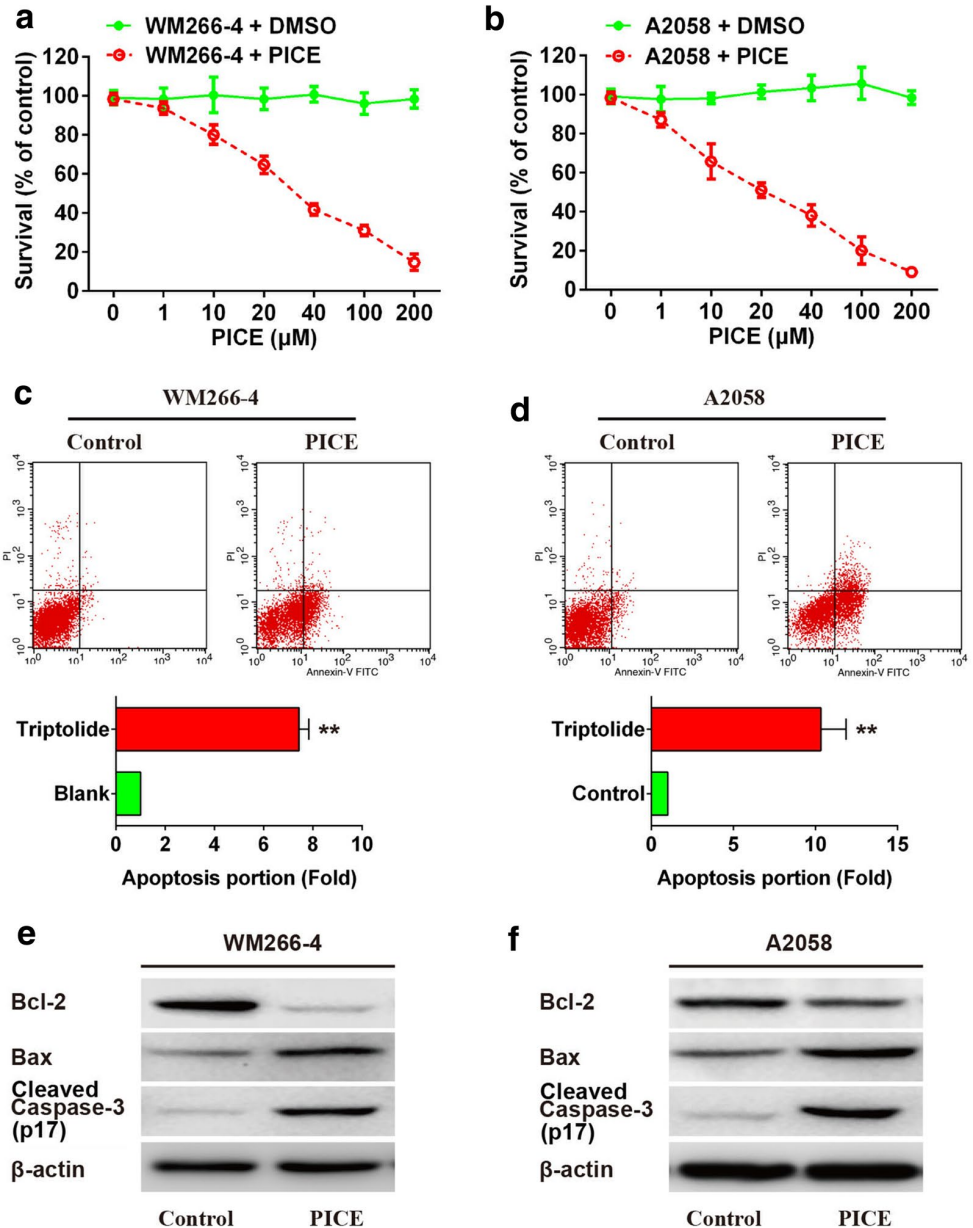
Piceatannol induces apoptosis of melanoma cells. (1) Effects of different concentrations of piceatannol on cell growth of WM266-4 (**a**) and A2058 (**b**); (2) Piceatannol increases apoptosis of WM266-4 (**c**) or A2058 (**d**) cells, as measured by flow cytometry; (3) Protein level of Bcl-2, Bax and activated caspase-3 (p17) in WM266-4 (**e**) and A2058 (**f**) cells incubated with piceatannol, as demonstrated by western blot. *P < 0.05, **P < 0.01 compared to blank control

To investigate the role of piceatannol on the growth of melanoma cells, WM266-4 and A2058 cells were treated with piceatannol at different concentration for 36 h, and then the cell activity was determined by MTT Assay. The result was shown in the Fig. 1a, the growth of WM266-4 cells was restrained with an IC_50_ of 29.4 μM piceatannol in a dose-dependent manner whereas the growth inhibitory effect was observed with IC_50_ of 15.6 μM piceatannol in A2058 cell line (Fig. 1b). Thus, 30 μM of piceatannol concentration was used for the subsequent analysis.

To determine whether the decrease of cell vitality was related to cell apoptosis, we detected the apoptotic action of WM266-4 and A2058 cells treated with piceatannol using flow cytometry assay. In comparison with control group, apoptotic effect in WM266-4 and A2058 cells were notably increased with dose of 30 μM piceatannol treatment (Fig. 1c, d). It was needed to be further determined whether the piceatannol-induced apoptosis of melanoma cells was involved in the expression of pathway regulatory proteins, caspase-3, Bcl-2 and Bax. For this reason, western blot was conducted to test the expression change of these three proteins and we found that expression of caspase-3 and Bax was increased while the expression of Bcl-2 proteins was markedly decreased in the cells treated with piceatannol compared with control group (Fig. 1e, f).

### Piceatannol promotes expression of miR-181a

**Fig. 2:**
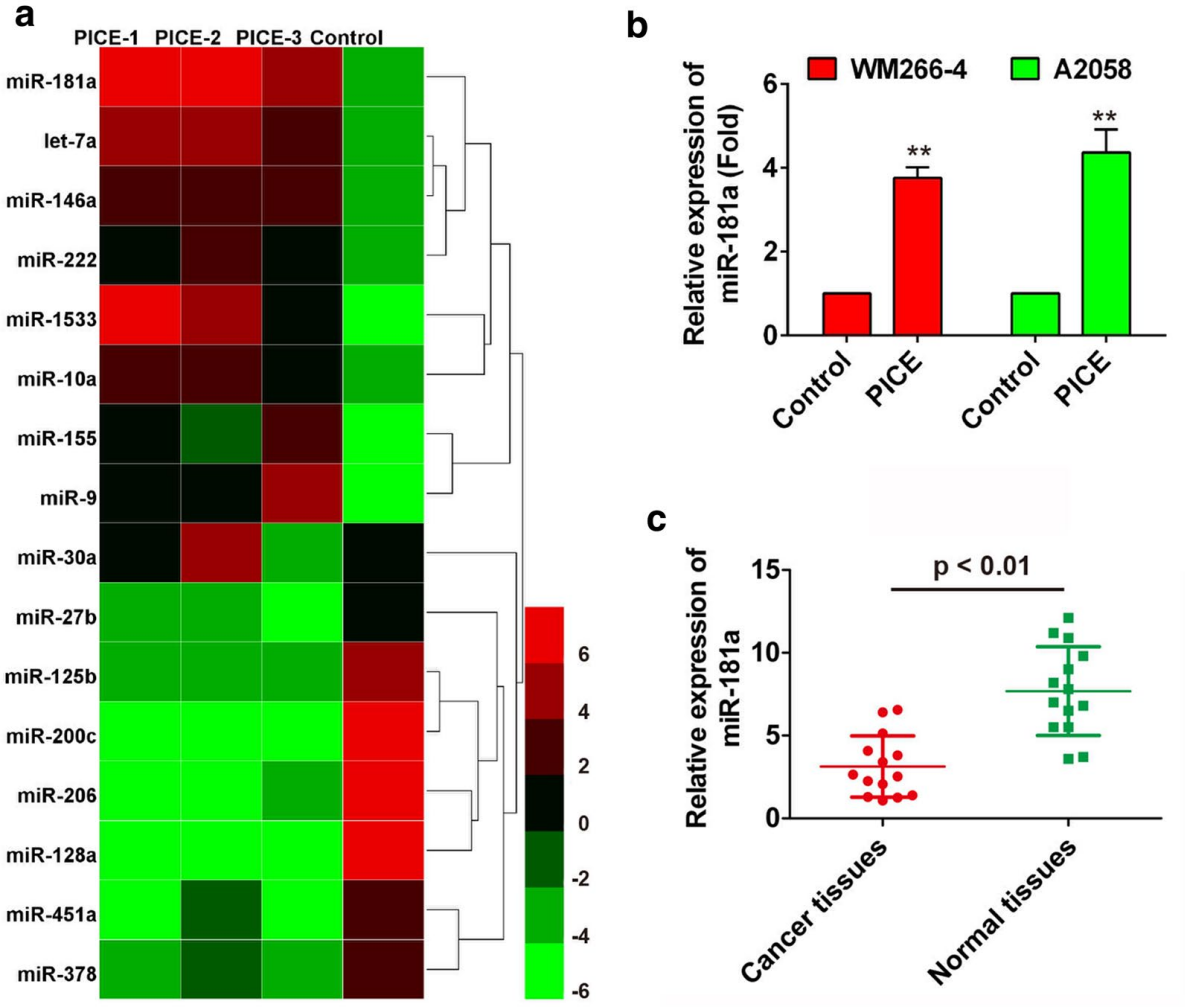
Piceatannol promotes the expression of miR-181a. **a** Unsupervised analysis heat map of 4 different samples. All samples are underwent cell clustering, and the top 16 microRNAs with highest standard deviation (SD  >  1) are enlisted. Each column represents one specimen and each row represents one microRNA. The color scale from 6 to − 6 shown at the right bottom of color map indicates the relative expression of a miRNA in all subjected samples: green color represents expression level lower than control while red color represents an expression level higher than control, 8 up-regulated and 8 down-regulated miRNAs were shown on the map; **b** Relative expression of miR-181a with and without piceatannol treatment in WM266-4 and A2058 cells; **c** miR-18a relative expression level in cancer tissues compared with normal tissues was analyzed using qRT-PCR. *P < 0.05, **P < 0.01 compared to normal control

Compared to their respective controls, the differentially expressed miRNAs in treatment group have been identified and classified into up-regulated and down-regulated groups, in accordance with the principle that ratio should be more than 2 comparing with control. It was shown that miR-181a was remarkably upregulated providing a reason to conduct further investigation on this miRNA (Fig. 2a). To validate the screened out result by PCR array, the expression of miR-181a was quantified by real-time RT-PCR. The result showed that the level of miR-181a was increased in both WM266-4 and A2058 cells treated with piceatannol for 48 h (Fig. 2b). To confirm the previous result of miR-181a accurately, 10 melanoma tissues and 10 normal tissues were subjected to quantitative RT-PCR for measuring the expression of miR-181a in tissues. The expression of miR-181a was remarkably decreased in melanoma tissues than in normal tissues (Fig. 2c).

### Bcl-2 is a direct target of miR-181a in melanoma cells

**Fig. 3:**
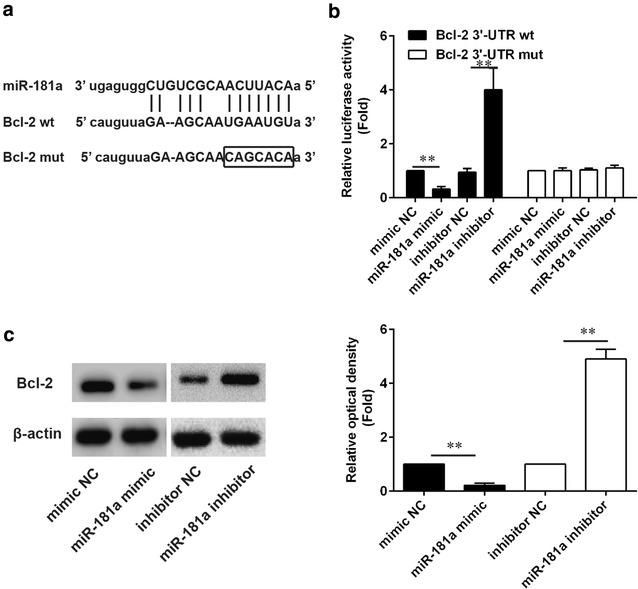
Bcl-2 is the direct target of miR-181a. **a** Schematic representation of Bcl-2 mRNA with a putative 3ʹ-UTRmiR-181a-binding site and sequences of wildtype (Bcl-2 WT) and mutant (Bcl-2mut) miR-181a target sites. Seed region is framed; **b** Luciferase activity of 3ʹ-UTR-Bcl-2 (wt) was inhibited with Transfection Of miR-181a and such inhibition was not present in the miR-181a-binding site (mut) with mutations or with participation of miR-181a inhibitor. The influence of miR-181a on expression level of Bcl-2 was compared to the mimic negative control; **c** Western blotting analysis of Bcl-2 and β-actin protein levels after transfection of miR-181a or mimic NC, or miR-181a inhibitor or inhibitor NC. **P < 0.01

Bcl-2 is a one of the regulatory molecules for the cell apoptosis. The putative miR-181a binding site was identified within the 3ʹ-UTR of Bcl-2 mRNA by Target Scan analysis (Fig. 3a). To test the direct binding of miR-181a to this site, wild type or mutant full length Bcl-23ʹ-UTR luciferase reporter constructs were generated. The recombinant Bcl-23ʹ-UTR constructs were transfected into HEK cells together with miR-181a mimic or miR-181 inhibitor. We observed that in HEK cells transfected with Bcl-2 wild type constructs, the luciferase activity was significantly inhibited by miR-181a compared to the controls, indicating that miR-181a can directly interact with the 3ʹ-untranslated region of Bcl-2 mRNA. Plus, miR-181a did not restrain the luciferase activity of Bcl-2 with mutations in the miR-181a-binding site **(**Fig. 3b). The western blot analysis was conducted to determine protein expression of Bcl-2 and we uncovered that miR-181a decreases the Bcl-2 protein in HEK cells compared to the negative control miRNA while the Bcl-2 protein expression was increased with miR-181a inhibitor (Fig. 3c). These results indicated that miR-181a directly targets the Bcl-2 3ʹ-UTR to suppress Bcl-2 protein translation.

### Silencing of miR-181a mitigates the pro-apoptotic effects of piceatannol in melanoma cells

**Fig. 4:**
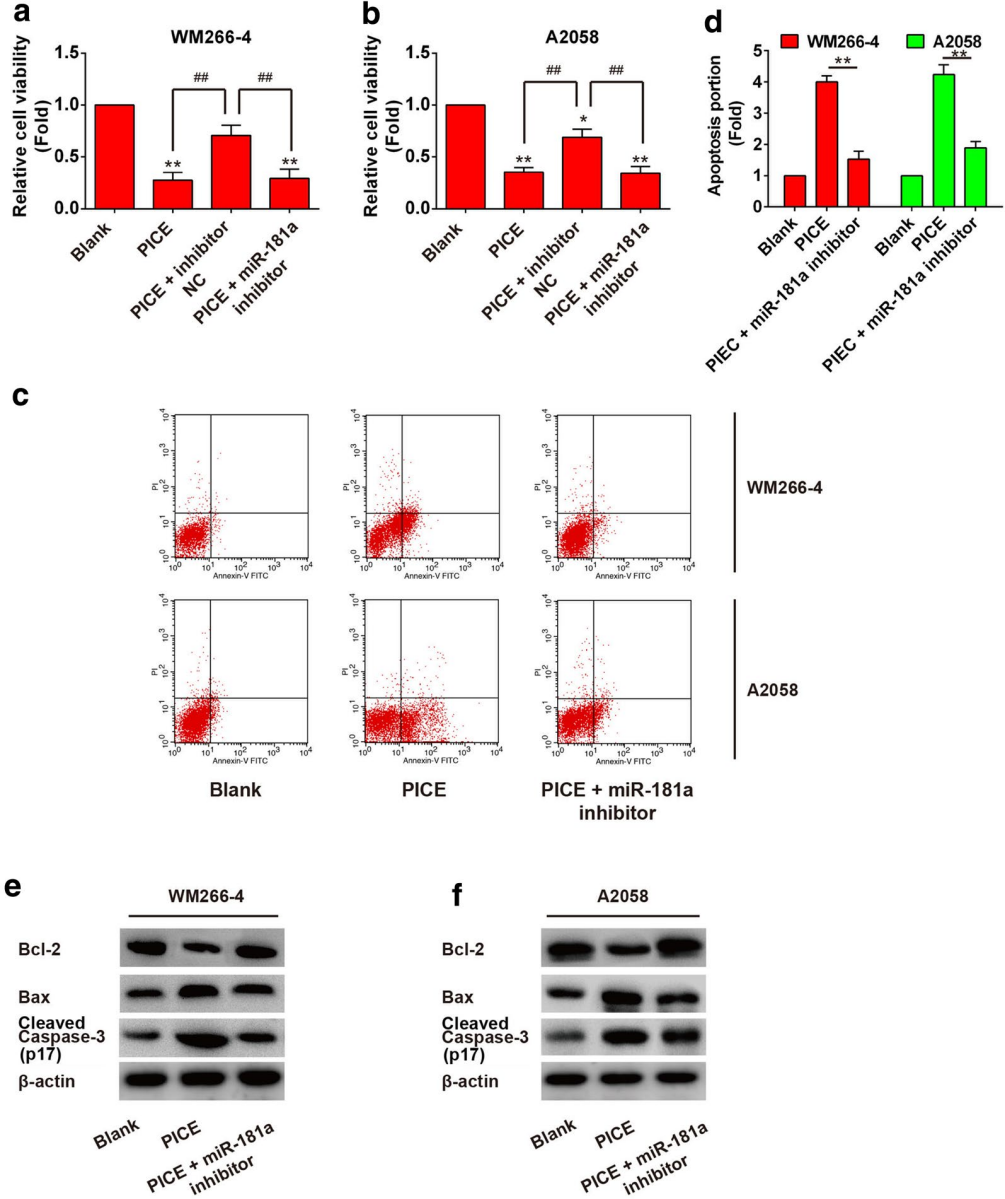
miR-181a knock down reduces the apoptotic effect of piceatannol on melanoma cells. (1) Cell viability assay (MTS) of WM266-4 (**a**) and A2058 (**b**) cells transfected with miR-181a or miR-181a inhibitor after piceatannol treatment; (2) Silencing of miR-181a alleviates the fall of cell apoptotic activity induced by piceatannol in WM266-4 (**c**) and A2058 (**d**) cells; (3) Inhibition of miR-181a stimulates expression dysregulation of activated caspase-3, Bax and Bcl-2 proteins in WM266-4 (**e**) and A2058 (**f**) cells. *P < 0.05, **P < 0.01 compared to normal control, ##*P* < 0.01

To determine whether the overexpression of miR-181a is participate in the piceatannol-induced apoptosis in melanoma cells, transfected specific inhibitor was used to knockdown the miR-181a, and we monitored the change of apoptotic action in melanoma cells. As shown in Fig. 4a, b, miR-181a inhibitor reinstated the reduction of cell vitality induced by piceatannol and it decreased the apoptotic role of piceatannol in melanoma WM266-4 and A2058 cells (Fig. 4c, d). The dysregulation of Bax, caspase-3, and Bcl-2 caused by piceatannol was reversed by miR-181a inhibitor in WM266-4 and A2058 cells (Fig. 4e, f).

## Discussion

In this study, we have demonstrated that piceatannol restrains the growth of melanoma cells. The expression of miR-181a was promoted by piceatannol and it caused up-regulation of Bax, down-regulation of Bcl-2 and activation of caspase-3, ultimately induced the apoptosis in melanoma cells. Above all, we discovered that highly expressed miR-181a induces apoptosis in melanoma cells, and pro-apoptotic action of piceatannol could be reduced by knockdown of miR-181a. Overall, our work demonstrated the pro-apoptotic effect of piceatannol in melanoma cells and disclosed the contribution of miR-181a in this biological process. And, present study suggests that piceatannol could be a new therapeutic agent for treatment of melanoma. This study is the first to report that promotion of miR-181a induced by piceatannol offers a therapeutically beneficial option for melanoma treatment, as evidenced by using clinical samples and cell lines and analysis.

Resveratrol and its derivative piceatannol specifically cause cell death in cancer cells [20–22]. Because of their greatly enriched ER-mitochondria tethering, cancer cells are highly susceptible for resveratrol/piceatannol-induced reduction of SERCA (sarco/endoplasmic reticulum Ca^2+^ ATPase) activity to yield mitochondrial Ca^2+^ overload and subsequent cancer cell death [23]. Among trans-resveratrol analogs, such as pterostilbene, piceatannol, polydatin and oxyresveratrol, was the more promising candidate for future studies regarding treatment of leishmaniasis [24]. A number of studies showed that piceatannol participate in inhibition of biological activities, such as invasion, progression, metastasis and drug-resistance, in lung cancer cells [25], colorectal cancer [26], breast cancer [27], ovarian cancer [28], prostate cancer [29] and leukemia [30]. However, few reports have been made on the effects of piceatannol in melanoma. Piceatannol was investigated for its anti-oxidative property and ability to inhibit melanogenesis to be used as new skin lightening agent [31]. A study investigated the anti-melanoma properties of dietary piceatannol considering its potent pro-apoptotic capacity at low concentration [9]. However, potential of piceatannol as new agent in melanoma treatment has been over looked. Our results emphasize this anti-cancer role of piceatannol by exhibiting that the capability of piceatannol to restrain melanoma cells growth by partially influencing Bax, Bcl-2 and caspase-3 expression in apoptosis, suggesting that piceatannol could be used for the treatment of melanoma.

A mRNA can be targeted by multiple miRNAs, and, conversely, a miRNA can interact with multiple mRNAs [32]. Recent experimental and clinical studies have uncovered that melanoma is influenced by various miRNAs, for instance miR-138, miR-211, miR-18b, miR-339-3p, miR-542-3p and so on [33–37]. In accordance with our microarray analysis, miR-181a is distinctively overexpressed in piceatannol-treated cells compared with normal control (Fig. 2a). Therefore, we selected miR-181a as a new candidate miRNA promotes apoptosis in melanoma cells to conduct further investigation. miR-181a has previously been reported to be upregulated in diverse cancers, such as ovarian cancer [38], gastric cancer [39], leukemia [40], and be downregulated in breast cancer [41], regulating epithelial-to-mesenchymal transition, cell proliferation, migration andG1/S transition. Yet, the exact function of miR-181a in the regulation of the apoptotic effect of piceatannol in melanoma cells remains unclear and this study is first to report its regulatory function with systematic analysis. In this study, we found that miR-181a dramatically overexpressed in both WM266-4 and A2058 cells treated with piceatannol, and downregulated in melanoma cancer tissues compared with its adjacent ones. Thus we suggest that, high level of miR-181a expression might associate with apoptotic effect of piceatannol in melanoma cells.

Bcl-2 is known to be a crucial anti-apoptotic protein and miRNA mediated Bcl-2 suppression has been demonstrated in a wide variety of cancers [42–44]. miR-181a was one of the miRNAs that downregulated in post-transcriptional level and preserve the low expression of relative proteins, such as silent mating-type information regulation 2 homologue 1 (SIRT1), which regulates various biological processes including transcription, apoptosis and muscle differentiation by deacetylating key proteins [45]. As estimated to be potent regulator in cell apoptosis by specifically targeting Bcl-2, miR-181a has been subjected to diverse level of tests in many different cancers and its functions are well documented [46]. A research presented evidence that in vivo allergic inflammation promotes the metastatic potential of mouse melanoma cells and miR-181a would be a valuable target for the development of anti-allergic drugs [47]. Several pieces of evidence suggested that the diminished expression of miR-181a in superficial spreading melanoma may contribute to the onset of melanoma subtype [48]. However, to date underlying mechanism of interaction between miR-181a and Bcl-2 in melanoma cells has not been fully understood and here we investigated into miRNA-protein interaction by defining specific binding sites in melanoma cells, and our findings would provide some insights into mechanism of anti-cancer effect of piceatannol. In accordance with the data presented in this study, it is shown that Bcl-2 is a direct target of miR-181a, via an independent binding site in its 3′-UTR, which reduces its expression, activity and downstream signaling, and our functional experiments validate Bcl-2 as a critical mediator of the pro-apoptotic effects of miR-181a in melanoma cells. The knockdown results of miR-181a contribute to make a further validation of these data. However, our data do not address whether miR-181a is involved in melanoma progression and invasion.

## Conclusions

To sum up, our findings showed that piceatannol promoted the expression to miR-181a, which mediated pro-apoptotic effect of piceatannol by suppressing Bcl-2 in melanoma cells. It provides basic information to better understanding the molecular mechanism involved in the anti-cancer activity of piceatannol in melanoma cells and suggested that piceatannol could be a potent agent for melanoma treatment. To best of our knowledge, this study is the first to report that systematically investigated the anti-cancer effect of piceatannol by probing into its mediatory miRNA for melanoma cells. It is needed to conduct further investigation on validation of therapeutic functions of piceatannol for melanoma in vivo, and should be tested against melanoma in clinical practice.

## Abbreviations

CCK-8: Cell Counting Kit-8
OD: optical density
FDP: false discovery rate of P value
SIRT1: silent mating-type information regulation 2 homologue 1
